# Synephrine potentiates lidocaine‐induced cutaneous analgesia via α‐adrenergic receptors in male rats

**DOI:** 10.14814/phy2.70758

**Published:** 2026-03-26

**Authors:** An‐Kuo Chou, Chong‐Chi Chiu, Kuo‐Sheng Liu, Yu‐Wen Chen, Ching‐Hsia Hung, Jhi‐Joung Wang

**Affiliations:** ^1^ Department of Anesthesiology China Medical University Hospital Taichung Taiwan; ^2^ School of Medicine, College of Medicine China Medical University Taichung Taiwan; ^3^ Department of General Surgery & Department of Medical Education and Research E‐Da Cancer Hospital, I‐Shou University Kaohsiung Taiwan; ^4^ School of Medicine, College of Medicine I‐Shou University Kaohsiung Taiwan; ^5^ Department of Pharmacy Chia Nan University of Pharmacy and Science Tainan Taiwan; ^6^ Department of Medical Research Chi Mei Medical Center Tainan Taiwan; ^7^ Department of Physical Therapy, College of Health Care China Medical University Taichung Taiwan; ^8^ Department of Physical Therapy, College of Medicine National Cheng Kung University Tainan Taiwan; ^9^ Institute of Allied Health Sciences, College of Medicine National Cheng Kung University Tainan Taiwan; ^10^ Department of Anesthesiology Tri‐Service General Hospital & National Defense Medical Center Taipei Taiwan

**Keywords:** infiltrative cutaneous analgesia, isobolograms, phentolamine, phenylephrine, synephrine

## Abstract

This study aimed to assess the cutaneous analgesic effect of the lidocaine–synephrine combination and its underlying α‐adrenergic receptor‐mediated mechanism in comparison with phenylephrine. Cutaneous analgesia after subcutaneous drug injection was assessed by inhibition of the cutaneous trunci muscle reflex in response to needle pinpricks on the shaved dorsal skin of Sprague–Dawley rats. Synephrine, like the local anesthetic lidocaine, is capable of inducing cutaneous analgesia but is less potent. On an equipotent basis (ED_25_, ED_50_ [50% effective dose], and ED_75_), the block duration induced by synephrine or phenylephrine was longer than that induced by lidocaine (*p* < 0.01). When combined with lidocaine, the ED_50_ for synephrine decreased from 326 (183–579) μmol/kg to 141 (128–156) μmol/kg (*p* < 0.01), and for phenylephrine, it decreased from 15.8 (9.0–27.9) μmol/kg to 6.2 (5.9–6.6) μmol/kg (*p* < 0.01). Lidocaine (ED_95_) in combination with either synephrine (52 μmol/kg) or phenylephrine (1.6 μmol/kg) prolonged the duration of action (*p* < 0.001), while phentolamine (0.3 μmol/kg) reversed these effects. We concluded that synephrine produces dose‐dependent cutaneous analgesia that is less potent but longer‐lasting than lidocaine. The enhanced analgesic effects of the lidocaine–synephrine and lidocaine–phenylephrine combinations are mediated through α‐adrenergic receptor activation.

## Graphical abstract

The enhanced analgesic effects of lidocaine–synephrine or lidocaine–phenylephrine combinations are mediated via α‐adrenergic receptor activation.

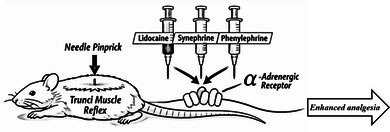

## 1 INTRODUCTION

Synephrine, an active constituent of Citrus aurantium, has been investigated for its potential therapeutic and health‐promoting effects (Perova et al., 2021). Synephrine is synthesized from octopamine through the enzymatic activity of phenylethanolamine N‐methyltransferase (Stohs et al., 2020). Recent studies have shown that octopamine produces dose‐dependent local anesthetic effects (Chou et al., 2025). A major limitation of local anesthetics is their relatively short duration of action, which substantially reduces their utility in prolonged surgical or interventional procedures (Li et al., 2019). Bupivacaine provides prolonged local anesthesia; however, its clinical application is constrained by potential cardiotoxic effects (Hino et al., 2020). To enhance the efficacy and safety of local anesthetics, epinephrine is commonly incorporated as an adjuvant. Through α/β‐adrenergic receptor activation, epinephrine induces vasoconstriction, which limits local blood flow, reduces systemic uptake of the anesthetic, and extends its pharmacological action (Becker & Reed, 2012; Schnabl et al., 2021). While phenylephrine (m‐synephrine) demonstrates selective affinity for α1‐adrenergic receptors (Richards et al., 2024), synephrine engages with various adrenergic receptor subtypes, reflecting a less selective binding profile (Stohs et al., 2020).

Clinical application of local anesthetics typically requires administration at comparatively high doses to ensure sufficient anesthetic or analgesic efficacy. However, when adjuvants are used to potentiate their effects, the additional benefit may be underestimated due to a ceiling effect already reached by the local anesthetic alone. Isobolographic analysis is an accepted method for evaluating drug interactions by combining two agents at fixed ED_50_ (50% effective dose) doses; this technique facilitates the determination of whether the drug combination exhibits additive, antagonistic, or synergistic effects (Tallarida, 2006). Phentolamine, characterized as a nonselective α‐adrenergic receptor antagonist, was initially developed and clinically applied for the reversal of soft tissue anesthesia (Daublander et al., 2017; Nourbakhsh et al., 2012). Furthermore, administration of phentolamine prior to patient discharge has been reported to prevent finger necrosis following soft tissue anesthesia with lidocaine in combination with epinephrine (Zhang et al., 2017). Our previous investigation utilized phentolamine to inhibit the potentiating effects of epinephrine on local anesthetic action (Chou et al., 2019).

This study aimed to evaluate the cutaneous analgesic effect of combining lidocaine with synephrine, to compare its efficacy with that of the phenylephrine–lidocaine combination, and to investigate its mechanism of action (α‐adrenergic receptors).

## 2 MATERIALS AND METHODS

### 2.1 Animals and ethical statement

The Institutional Animal Care and Use Committee of Chi Mei Medical Center (Taiwan) approved the protocol (IACUC no. 110122301). Male Sprague–Dawley rats (210–260 g) were obtained from BioLASCO, Taipei, Taiwan. The animals were housed in a temperature‐controlled environment under a 12‐h light/12‐h dark cycle (lights on from 6:00 am to 6:00 pm), with ad libitum access to food and water. Animals were fed a standard laboratory rodent chow (LabDiet 5001, PMI Nutrition International, St. Louis, MO; catalog no. 5001).

### 2.2 Drugs

Lidocaine hydrochloride (cat. no. 1366013, United States Pharmacopeia (USP) Reference Standard, Sigma‐Aldrich, St. Louis, Missouri, USA), phenylephrine hydrochloride (cat. no. BP284, British Pharmacopoeia (BP) Reference Standard, Sigma‐Aldrich), synephrine (cat. no. 1642609, USP Reference Standard, Sigma‐Aldrich), and phentolamine mesylate (cat. no. 1530004, USP Reference Standard, Sigma‐Aldrich) were completely dissolved in 0.6 mL of saline prior to injection.

### 2.3 Cutaneous analgesia

Each animal underwent a 3‐day environmental acclimatization period before the start of the experiment. Then, a total volume of 0.6 mL of drug solution was injected subcutaneously into the shaved dorsal skin of the rats using a 30 G needle‐syringe. The injection area, within a 2‐cm diameter circle (wheal), was then marked with an ink pen. The wheal was stimulated with an 18‐gauge needle attached to a *von* Frey filament (Chou et al., 2023). The analgesic effect was evaluated based on the drug's ability to block the cutaneous trunci muscle reflex (CTMR). CTMR is a polysynaptic spinal reflex characterized by a visible contraction of the dorsal cutaneous muscle in response to a noxious mechanical skin stimulus in rodents (Chou et al., 2024). Mechanical stimulation was applied using a standardized pinprick stimulus delivered perpendicular to the skin with sufficient force to elicit a clear CTMR response prior to drug administration. Baseline CTMR responsiveness was first confirmed by applying a pinprick stimulus outside the wheal area. To exclude potential central analgesic effects, subsequent pinprick stimuli were applied within the wheal area only after a positive CTMR response was observed outside the wheal. Each test consisted of six consecutive pinprick stimuli applied at intervals of approximately 3 s. CTMR blockade was defined as the absence of visible dorsal skin contraction following a stimulus. The degree of analgesia was expressed as the percentage of possible effect (%PE), calculated as the number of stimuli that failed to elicit a CTMR response divided by six, multiplied by 100. The duration was calculated from the time of subcutaneous injection to the full recovery of the CTMR. Dose–response data were analyzed by fitting a sigmoidal curve using nonlinear regression. The ED_50_ (50% effective dose) was determined from the fitted curve using SAS statistical software (SAS Institute, Cary, NC, USA) (Changchien et al., 2021; Minkin & Kundhal, 1999). ED_50_ is the dose required to achieve 50% of the desired therapeutic effect. Similarly, ED_25_, ED_75_, and ED_95_ represent the doses needed to produce 25%, 75%, and 95% of the therapeutic effect, respectively. The AUC (area under the curve) was calculated using Kinetica version 2.0.1 software (InnaPhase Corporation, Philadelphia, Pennsylvania) (Chiu et al., 2023).

### 2.4 Isobologram analysis of drug–drug interactions

Isobolographic analysis was used to evaluate ED_50_ values as equivalent doses, according to the method of Tallarida (2006). This method is widely applied in pharmacology to optimize drug combinations for enhanced therapeutic outcomes. First, dose–response curves for lidocaine, synephrine, and phenylephrine were constructed using eight animals per dose. Using isobolographic analysis, the theoretical ED_50_ for the combination of two drugs was determined. Then, dose–response curves for two‐drug combinations (e.g., synephrine and lidocaine) in fixed ratios were constructed, and the experimental ED_50_ was derived. Finally, the theoretical and experimental ED_50_ values were compared. If no statistically significant difference was found between the experimental and theoretical ED_50_, the interaction was classified as additive. Conversely, if the experimental ED_50_ was significantly lower than the theoretical ED_50_, the interaction was classified as synergistic.

### 2.5 Groups

Eight rats were randomly assigned to each dose group. In Group 1, dose–response curves of synephrine (163, 261, 522, 783, 1565, and 3130 μmol/kg), phenylephrine (1.6, 3.2, 6.4, 12.8, and 32.0 μmol/kg), and lidocaine (6.5, 13.0, 21.5, 41.7, and 57.4 μmol/kg) were constructed. Due to the similarity of the graphs, only the time course of the ED_75_ for synephrine and lidocaine was presented. The control group received 0.6 mL of saline solution. The block duration induced by synephrine was compared to that induced by lidocaine or phenylephrine based on equipotent doses (ED_25_, ED_50_, and ED_75_). The second group was analyzed for analgesic interactions between subcutaneous lidocaine in combination with either synephrine or phenylephrine. A time course was constructed for lidocaine at 2 × ED_50_, and for lidocaine (ED_50_) in combination with either synephrine (ED_50_) or phenylephrine (ED_50_). In Group 3, time courses of subcutaneous injection of lidocaine (ED_95_) alone, co‐injected with synephrine (52 μmol/kg), co‐injected with phenylephrine (1.6 μmol/kg), co‐injected with synephrine (52 μmol/kg) and phentolamine (0.3 μmol/kg), or co‐injected with phenylephrine (1.6 μmol/kg) and phentolamine (0.3 μmol/kg).

### 2.6 Statistical analysis

Values were expressed as mean ± SD or as ED_50_ with a 95% confidence interval. All data were analyzed using SPSS statistical software (version 17; IBM Corp., Armonk, NY, USA). A p‐value less than 0.05 was considered statistically significant. A Student's *t*‐test was used to compare the experimentally derived ED_50_ with the theoretical additive ED_50_. Other group differences were analyzed by one‐way or two‐way ANOVA (analysis of variance), followed by Tukey's HSD (honestly significant difference) post hoc test.

## 3 RESULTS

### 3.1 Synephrine‐induced cutaneous analgesia

**FIGURE 1:**
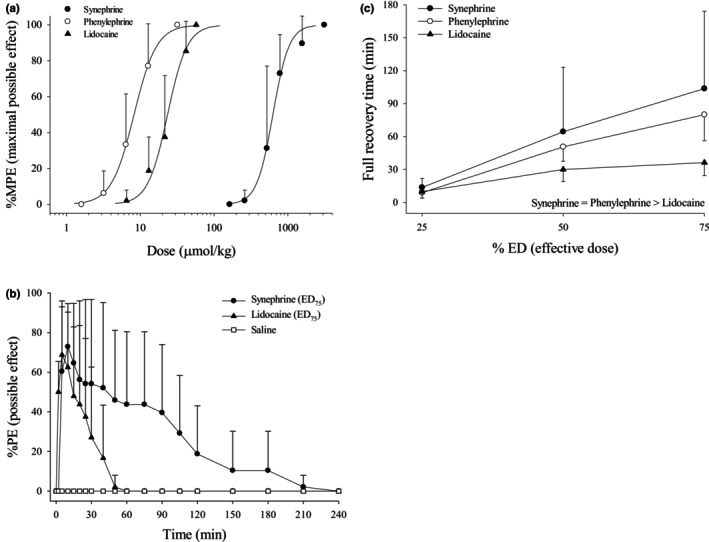
(a) Dose–response curves for infiltrative cutaneous analgesia following subcutaneous injection of synephrine, phenylephrine, and the local anesthetic lidocaine. (b) Time course of cutaneous analgesia after subcutaneous injection of synephrine (ED_75_), lidocaine (ED_75_), and saline (vehicle). (c) Duration of action of synephrine, phenylephrine, and lidocaine at equipotent doses (ED_25_, ED_50_, and ED_75_). Differences in duration were analyzed using two‐way ANOVA, followed by Tukey's HSD test for pairwise comparisons. Values are expressed as mean ± SD. Each group contains eight rats. ED_25_, 25% effective dose. ED_50_, 50% effective dose. ED_75_, 75% effective dose.

Subcutaneous injections of synephrine, phenylephrine, and the local anesthetic lidocaine produced dose‐dependent cutaneous analgesic effects (Figure 1a). The ED_25_, ED_50_, ED_75_, and ED_95_ are shown in Table 1. The rank order of potency (ED_50_) is phenylephrine > lidocaine > synephrine (*p* < 0.01; Table 1). Due to the similarity in the effects of these test drugs, only synephrine and lidocaine were presented (Figure 1b). At ED_75_, synephrine and lidocaine induced sensory/nociceptive blockade of 73% and 69%, respectively (Figure 1b). No sensory/nociceptive blockade was produced by subcutaneous injection of saline (Figure 1b). The block duration induced by synephrine or phenylephrine was longer than that induced by lidocaine, as indicated by the ED_25_, ED_50_, and ED_75_ values (*p* < 0.01; Figure 1c).

**TABLE 1:** Derivation of effective doses for cutaneous analgesia.

Drug (μmol/kg)	ED_50_ (95% CI)	ED_25_	ED_75_	ED_95_
Synephrine	628 (555–711)	476	828	1318
Phenylephrine	8.2 (7.1–9.5)	5.6	12.1	23.2
Lidocaine	23.5 (20.8–26.4)	16.7	32.9	58.1

*Note*: Effective doses (EDs) were derived from dose–response curves of cutaneous analgesia produced by synephrine, phenylephrine, and lidocaine. ED values were calculated using the NLIN procedure in SAS (SAS Institute Inc., Cary, NC, USA). The rank order of potency (ED_50_) was phenylephrine > lidocaine > synephrine (*p* < 0.01, one‐way ANOVA followed by Tukey's HSD test for pairwise comparisons).

Abbreviations: 95% CI, 95% confidence interval. ED_25_, 25% effective dose. ED_50_, 50% effective dose. ED_75_, 75% effective dose. ED_95_, 95% effective dose.

### 3.2 Synergistic analgesic effects of the lidocaine–synephrine combination

**FIGURE 2:**
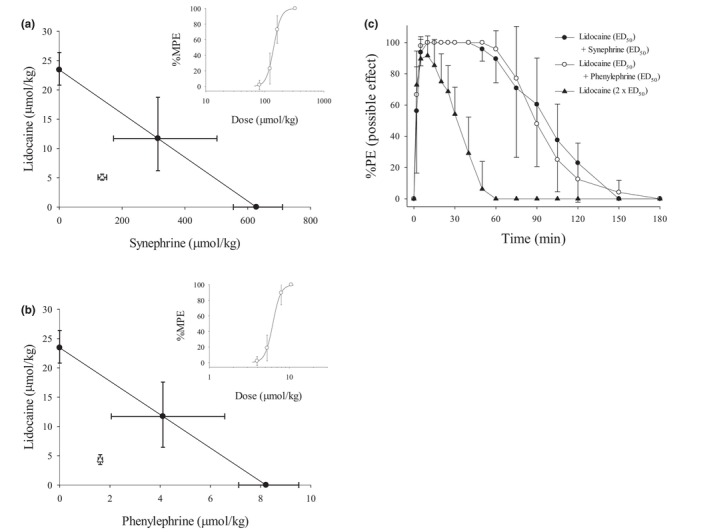
(a, b) Isobolograms for the analysis of drug–drug interactions. The dots on the X‐ and Y‐axes represent the ED_50_ values of the individual drugs, and the line connecting the axes represents the theoretical additive line. The hollow triangle (△) indicates the experimental ED_50_ with a 95% confidence interval, whereas the filled circle (●) indicates the theoretical ED_50_ with a 95% confidence interval. The dose–response curves for lidocaine co‐injected with synephrine and phenylephrine are shown in the upper right corners of panels a and b, respectively. (c) Time course of cutaneous analgesia after subcutaneous injection of lidocaine (2 × ED_50_) alone or lidocaine (ED_50_) in combination with either synephrine (ED_50_) or phenylephrine (ED_50_). Values are expressed as mean ± SD. Each group contains eight rats. ED_50_, 50% effective dose.

Dose–response curves for the two‐drug combinations at fixed ratios were constructed (Figure 2a,b). The theoretical additive lines and experimentally derived ED_50_ values are shown in Figure 2a,b. After isobolographic analysis, significant differences between the theoretical and experimentally derived ED_50_ values were observed in the lidocaine–synephrine and lidocaine–phenylephrine combinations (*p* < 0.01; Table 2), both indicating synergistic interactions. Lidocaine (ED_50_) mixed with either synephrine (ED_50_) or phenylephrine (ED_50_) increased blocking potency (%MPE) over lidocaine (2 × ED_50_) (Figure 2c). The AUC and full recovery time of lidocaine (ED_50_) injected in combination with either synephrine (ED_50_) or phenylephrine (ED_50_) were greater than those of lidocaine (2 × ED_50_) injected alone (*p* < 0.001; Table 3).

**TABLE 2:** Isobologram analysis of drug–drug interactions.

Drug combination (μmol/kg)	ED_50_ (95% CI)	
	Experimental value	Theoretical value
Lidocaine + Synephrine	141 (128–156)**	326 (183–579)
Lidocaine + Phenylephrine	6.2 (5.9–6.6)**	15.8 (9.0–27.9)

*Note*: Values are expressed as ED_50_ with a 95% CI.

Abbreviations: 95% CI, 95% confidence interval; ED_50_, 50% effective dose.

** *p* < 0.01, compared with the theoretical value (Student's *t*‐test).

**TABLE 3:** The percent of maximal possible effect (%MPE), duration, and area under the curves (AUCs).

	%MPE	Duration (min)		AUCs (%MPE × min)
		Complete block time	Full recovery time	
*Lidocaine + Adjuvants*				
Lidocaine (2 × ED_50_)	92 ± 13	―	49 ± 6	2699 ± 647
Lidocaine (ED_50_) + Synephrine (ED_50_)	100 ± 0	60 ± 22	122 ± 33^a^	8792 ± 2318^a^
Lidocaine (ED_50_) + Phenylephrine (ED_50_)	100 ± 0	64 ± 12	120 ± 38^a^	8732 ± 2123^a^
*Lidocaine + Adjuvants + Phentolamine*				
Lidocaine (ED_95_)	96 ± 8	―	48 ± 10	2722 ± 530
Lidocaine (ED_95_) + Synephrine (52 μmol/kg)	100 ± 0	74 ± 20	135 ± 35^b^	9950 ± 2554^b^
Lidocaine (ED_95_) + Synephrine (52 μmol/kg) + Phentolamine (0.3 μmol/kg)	100 ± 0	18 ± 11	49 ± 4	3115 ± 522
Lidocaine (ED_95_) + Phenylephrine (1.6 μmol/kg)	100 ± 0	71 ± 11	135 ± 31^b^	9930 ± 1467^b^
Lidocaine (ED_95_) + Phenylephrine (1.6 μmol/kg) + Phentolamine (0.3 μmol/kg)	98 ± 6	14 ± 10	54 ± 12	3274 ± 762

*Note*: The cutaneous analgesic effect of subcutaneous injection of lidocaine combined with either synephrine or phenylephrine, with or without phentolamine, was evaluated. Values are expressed as mean ± SD. The symbol (a) indicates *p* < 0.001 compared with lidocaine (2 × ED_50_); the symbol (b) indicates *p* < 0.001 compared with lidocaine (ED_95_) using one‐way ANOVA, followed by Tukey's HSD test for pairwise comparisons.

### 3.3 Effect of phentolamine on the lidocaine–synephrine combination

**FIGURE 3:**
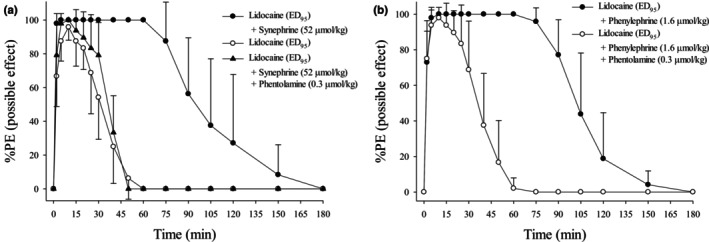
(a, b) Time course of cutaneous analgesia after subcutaneous injection of lidocaine (ED_95_) alone, co‐injected with synephrine (52 μmol/kg), co‐injected with phenylephrine (1.6 μmol/kg), co‐injected with synephrine (52 μmol/kg) and phentolamine (0.3 μmol/kg), or co‐injected with phenylephrine (1.6 μmol/kg) and phentolamine (0.3 μmol/kg). Values are expressed as mean ± SD. Each group contains eight rats. ED_95_, 95% effective dose.

Lidocaine at ED_95_ resulted in a 96% inhibition of sensory/nociceptive response (Figure 3a). Synephrine (52 μmol/kg) or phenylephrine (0.3 μmol/kg) enhanced the effect of lidocaine at its ED_95_ dose (Figure 3a,b). The AUCs and full recovery time of lidocaine (ED_95_) co‐injected with either synephrine (52 μmol/kg) or phenylephrine (1.6 μmol/kg) were greater than those of lidocaine (ED_95_) alone (*p* < 0.001; Table 3). There were no significant differences in the addition of phentolamine (0.3 μmol/kg) to the synephrine–lidocaine or phenylephrine–lidocaine combination compared to lidocaine (ED_95_) alone (Table 3).

## 4 DISCUSSION

The present study demonstrates that synephrine produces dose‐dependent cutaneous analgesia, providing new evidence for a peripheral sensory modulatory role of this adrenergic compound (Stohs et al., 2020). When administered at equipotent doses, synephrine and phenylephrine produced a longer duration of cutaneous blockade than lidocaine, suggesting differences in drug disposition or receptor‐mediated modulation of peripheral nerve function (Lirk et al., 2018). Adrenergic regulation of peripheral nociceptive transmission has been shown to modulate sensory thresholds through both vascular and neuronal mechanisms (Millan, 2002). Isobolographic analysis further showed that lidocaine combined with either synephrine or phenylephrine resulted in synergistic interactions, implying that these agents modulate analgesic efficacy through complementary mechanisms (Tallarida, 2006). The enhancement of lidocaine‐induced analgesia by synephrine and phenylephrine, and its reversal by phentolamine, supports the involvement of α‐adrenergic receptor‐mediated processes (Hersh et al., 2017).

Previous studies have reported analgesic and antispasmodic effects of Citrus aurantium extracts in both traditional and experimental contexts, suggesting the presence of bioactive compounds capable of modulating nociceptive signaling (Ammar et al., 2012; Choi et al., 2014). Synephrine, a naturally occurring protoalkaloid structurally related to octopamine, has been proposed as one of the major contributors to these biological effects (Stohs et al., 2020). Phytochemical‐derived adrenergic agonists have previously been shown to influence peripheral sensory processing via modulation of sympathetic‐sensory coupling (Pertovaara, 2006). In support of this hypothesis, recent evidence demonstrates that subcutaneous administration of octopamine produces local anesthetic‐like activity, indicating that structurally related adrenergic compounds may influence peripheral nerve excitability (Chou et al., 2025). Because inhibition of voltage‐gated sodium channels is a fundamental mechanism underlying local anesthetic action, these findings suggest a plausible physiological basis for synephrine‐induced sensory blockade (Lirk et al., 2018). Adrenergic modulation has also been shown to indirectly affect sodium channel availability by altering local tissue perfusion and inflammatory signaling (Gold & Gebhart, 2010). Although direct sodium channel blockade by synephrine has not been confirmed, the present results show that synephrine produced a degree of sensory inhibition comparable to lidocaine at the ED_75_ level, indicating functional relevance at the tissue level (Figure 1b).

Synephrine produced dose‐dependent cutaneous analgesia but exhibited lower potency than lidocaine, whereas phenylephrine demonstrated greater potency despite sharing structural similarities, indicating divergence in receptor affinity or downstream signaling efficiency among these adrenergic agents (Table 1). Such variability is consistent with prior pharmacological characterizations of adrenergic agonists, which demonstrate compound‐specific differences in receptor selectivity and efficacy (Stohs et al., 2020). Differences in α1‐adrenergic receptor subtype activation have been shown to significantly influence vascular tone and peripheral nerve sensitivity (Docherty, 1998). Importantly, oral synephrine at commonly consumed doses has not been associated with significant cardiovascular adverse effects in humans, suggesting a physiological profile distinct from that of classical sympathomimetic agents (Suntar et al., 2018). In contrast, phenylephrine is widely used clinically for its vasoconstrictive properties, particularly in anesthesia‐related settings, highlighting functional differences despite structural similarity (Anusorntanawat et al., 2016). These differences highlight the need to consider both physiological efficacy and safety when evaluating adrenergic compounds for peripheral analgesic modulation (Stohs et al., 2020).

Although ultrashort spinal anesthesia is not routinely required, agents such as 2‐chloroprocaine are preferred when rapid offset is desired (Bhaskara et al., 2020). In this context, the longer duration of cutaneous blockade produced by synephrine compared with lidocaine suggests a potential physiological role in modulating the persistence of peripheral sensory inhibition (Figure 1c). Local vasoconstriction has been repeatedly shown to reduce systemic absorption of local anesthetics, thereby prolonging their peripheral action (Becker & Reed, 2012). Pharmacokinetic data indicate that synephrine has a relatively prolonged biological half‐life and undergoes extensive metabolism, factors that may contribute to sustained peripheral effects (Hengstmann & Aulepp, 1978). In addition, receptor binding dynamics, tissue distribution, and the potential formation of active metabolites may further influence the temporal profile of analgesia (Holford, 2017; Taehan Imsang Yangni, 2014). Together, these factors suggest that synephrine exhibits physiological properties distinct from classical local anesthetics (Stohs et al., 2020).

Isobolographic analysis provides a quantitative framework for assessing additive, antagonistic, or synergistic interactions between pharmacological agents acting on a shared physiological endpoint (Tallarida, 2006). In this study, the experimentally derived ED_50_ values for lidocaine combined with synephrine or phenylephrine fell below the theoretical additive line, indicating synergistic interactions at the level of cutaneous analgesia (Figure 2a,b). Such synergy implies that lower doses of each agent may be sufficient to achieve a given level of sensory blockade, potentially reducing off‐target effects (Tallarida, 2006). Synergistic interactions between vasoconstrictors and local anesthetics have been reported to improve both efficacy and safety margins (Uritu et al., 2025). These findings are consistent with previous reports showing that octopamine enhances lidocaine‐induced cutaneous analgesia, supporting a role for adrenergic modulation in peripheral nerve function (Chou et al., 2025). Given that both synephrine and phenylephrine activate α‐adrenergic receptors, vasoconstriction‐mediated reduction of local anesthetic clearance likely contributes to the observed enhancement of analgesic duration (Richards et al., 2024; Stohs et al., 2020).

Local anesthetics are rapidly absorbed into the systemic circulation, which limits the duration of peripheral nerve blockade and reduces sustained analgesic efficacy (Gasteiger et al., 2023; Saul et al., 2020). In such cases, the long‐acting local anesthetic bupivacaine can be used; however, it carries a risk of systemic toxicity (Bacon et al., 2019; On'Gele et al., 2024). The use of vasoconstrictive adjuvants to prolong local anesthetic action represents a well‐established physiological strategy (Bailard et al., 2014). Adrenergic adjuvants have been shown to reduce peak plasma concentrations of local anesthetics, thereby decreasing systemic toxicity risk (Flood et al., 2022). In the present study, co‐administration of lidocaine with synephrine or phenylephrine significantly prolonged cutaneous analgesia, paralleling findings observed with other adrenergic‐local anesthetic combinations (Zheng et al., 2023). These results support the concept that α‐adrenergic modulation can influence peripheral nociceptive processing and anesthetic duration (Richards et al., 2025).

Phenylephrine is a clinically established α‐adrenergic agonist widely used to manage spinal anesthesia‐induced hypotension (Dusitkasem et al., 2017). The reversal of the enhanced analgesic effect by phentolamine confirms that α‐adrenergic receptor activation is a key physiological mediator of the observed synergy (Hersh et al., 2017). The absence of cutaneous analgesia following administration of synephrine or phenylephrine alone at the tested doses suggests that these agents primarily modulate lidocaine pharmacokinetics rather than directly suppress nociceptive signaling (Nourbakhsh et al., 2012). This observation is consistent with evidence indicating that α‐adrenergic agonists alone exhibit limited direct antinociceptive effects at the peripheral level (Pertovaara, 2006).

Several limitations should be considered when interpreting these findings. Although synephrine has been reported to exhibit minimal subchronic toxicity in animal models and lacks cytotoxic effects in human hepatocytes, its potential neurotoxicity and systemic effects following peripheral administration remain insufficiently characterized (Arbo et al., 2009; Ribeiro et al., 2019). In addition, the present study did not directly assess cardiovascular or neurological safety outcomes, either alone or in combination with lidocaine. Further studies are therefore required to delineate the physiological safety profile and mechanistic pathways underlying synephrine‐mediated modulation of peripheral analgesia (Stohs et al., 2020).

## 5 CONCLUSION

In summary, synephrine produces dose‐dependent cutaneous analgesia and prolongs lidocaine‐induced sensory blockade through α‐adrenergic receptor–dependent mechanisms. Although less potent than lidocaine, synephrine and phenylephrine both extended the duration of analgesia and exhibited synergistic interactions when combined with lidocaine. These findings provide physiological evidence that adrenergic modulation can influence peripheral nociceptive processing and support further investigation into the mechanisms underlying adrenergic‐local anesthetic interactions.

## AUTHOR CONTRIBUTIONS

An‐Kuo Chou contributed to the conception and design of the study; acquisition, analysis, and interpretation of data; drafting of the manuscript; obtaining funding; and critical revision of the manuscript for important intellectual content. Chong‐Chi Chiu contributed to the acquisition, analysis, and interpretation of data; drafting of the manuscript; obtaining funding; and statistical analysis. Kuo‐Sheng Liu contributed to the analysis and interpretation of data; drafting of the manuscript; and statistical analysis. Yu‐Wen Chen contributed to the conception and design of the study; acquisition, analysis, and interpretation of data; drafting of the manuscript; obtaining funding; statistical analysis; critical revision of the manuscript for important intellectual content; and study supervision. Ching‐Hsia Hung contributed to the conception and design of the study; analysis and interpretation of data; drafting of the manuscript; critical revision of the manuscript for important intellectual content; statistical analysis; obtaining funding; administrative support; and study supervision. Jhi‐Joung Wang contributed to the conception and design of the study; acquisition and interpretation of data; drafting of the manuscript; and technical and material support.

## FUNDING INFORMATION

The grants from the National Science and Technology Council (NSTC 114‐2314‐B‐039‐038‐MY2; NSTC 114‐2314‐B‐039‐047‐), I‐Shou University (NCKUEDA11407), and the China Medical University (CMU114‐MF‐113), Taiwan.

## CONFLICT OF INTEREST STATEMENT

The authors have no conflict of interest to declare.

## ETHICS STATEMENT

The study protocol was reviewed and approved by the Ethics Committee for Animal Experimentation (IACUC no. 110122301).

## Data Availability

The datasets generated during and/or analyzed during the current study are available from the corresponding author on reasonable request.
